# Dataset on density functional theory investigation of ternary Heusler alloys

**DOI:** 10.1016/j.dib.2023.109971

**Published:** 2023-12-18

**Authors:** Ridwan Nahar, Ka Ming Law, Thomas Roden, Michael Zengel, Justin Lewis, Sujan Budhathoki, Riley Nold, Harshil Avlani, Babajide Akintunde, Naomi Derksen, Adam J. Hauser

**Affiliations:** Department of Physics and Astronomy, University of Alabama, AL, United States

**Keywords:** Density functional theory (DFT), Heusler alloys, Formation energy, Magnetic moment, Spin polarization, Density of states, Projected density of states

## Abstract

This paper contains data and results from Density Functional Theory (DFT) investigation of 423 distinct *X*_2_*YZ* ternary full Heusler alloys, where *X* and *Y* represent elements from the D-block of the periodic table and *Z* signifies element from main group. The study encompasses both “regular” and “inverse” Heusler phases of these alloys for a total of 846 potential materials. For each specific alloy and each phase, a range of information is provided including total energy, formation energy, lattice constant, total and site-specific magnetic moments, spin polarization as well as total and projected density of electronic states. The aim of creating this dataset is to provide fundamental theoretical insights into ternary *X*_2_*YZ* Heusler alloys for further theoretical and experimental analysis.

Specification Table

**Table:** 

Subject	Condensed Matter Physics
Specific Subject area	Density Functional Theory, Intermetallic compounds, Heusler Alloys
Data format	Excel, .dat
Type of data	Figures, Table, Collected data
Data collection	The first principal calculations were performed using Quantum Espresso [1] (Version 6.8) simulation package on *X*_2_*YZ* Heusler alloys. The total energy of each alloy was calculated as a function of the calculated lattice parameter. A Self-Consistent Field (SCF) calculation followed by a non-SCF calculation were performed to get the Density of States (DOS) and projected Density of States (PDOS) of each of these alloys. Spin Polarization ($P_{F})$ was calculated by using the density of states (DOS) of spin-up and -down electrons at Fermi energy (*E_F_*).
Data Source	Institution: The University of Alabama
Location	City, State: Tuscaloosa, Alabama
	Country: United States of America
Data accessibility	DOI:10.17632/by523ywzs9.3
	Direct URL to data: https://data.mendeley.com/datasets/by523ywzs9/3

## 1 Value of the Data

• This dataset will provide fundamental information about 423 ternary Heusler alloys in their regular (*L2_1_*) and inverse (*XA*) Heusler phases, for a total of 846 distinct alloys calculated. Structure, magnetic properties, and spin polarization values are provided as a launching point for future studies.
• This wide-scale database will serve as a valuable screening tool for identifying promising candidates and conducting thorough and comprehensive studies on ternary Heusler alloys.
• The dataset is also useful for machine learning studies of structure, phase stability, and electronic and magnetic properties of alloy systems.

## 2 Objective

Heusler alloys have attracted substantial attention within the research community due to their potential applications in fields such as spintronics [2] and thermo-electric devices [3]. Considerable focus is now being paid to identify novel candidates with advanced properties and their possible application in various fields. The enclosed comprehensive dataset of Heusler alloys, including structure properties and stability, magnetic structure and spin polarization, and electronic structure via density of states calculations, is valuable for researchers seeking to identify novel candidates and more quickly progress to subsequent in-depth theoretical and experimental exploration.

## 3 Data Description

Heusler compounds first attracted interest when Cu_2_MnAl was discovered by German scientist Fritz Heusler in 1903 [4]. This remarkable face-centered cubic compound exhibited ferromagnetic properties although none of its constituent elements possessed inherent ferromagnetism. Motivated by this discovery, researchers worldwide have identified over a thousand Heusler alloys and their relatives within the past century and the quest for more continues to this day. To facilitate this quest, we have generated collections of *X*_2_*YZ* Heusler alloys. This dataset stands out as one of the few comprehensive databases accessible for researchers in this field [5], offering a complementary source of data alongside the well-known Open Quantum Materials Datasets (OQMD) [6,7], Material Projects (MP) [8] and Automatic-FLOW for Materials Discovery (AFlow) [9]. Data on Materials Project (MP) are collected from two sources: (1) high-throughput calculations on supercomputing clusters; (2) academic community using MPContribs [10]. Data collected with (1) came from calculations performed using VASP [11], a well-reputed package that uses a full-potential method. However, self-consistent calculations (SCF) of many Heusler compounds and non-self-consistent calculations (NSCF) prior to DOS calculations were performed with a low k-point grid. Data for MP, OQMD and AFLOW were uploaded via Application Programming Interface (API) [12] from a supportive academic community comprise of many experienced researchers. However, the lack of a consistency in computation methods and parameters across over a thousand Heusler compounds makes it very difficult to create models of generalized behavior of Heuslers.

Moreover, MP, OQMD and AFLOW websites contain only a subset of Heusler compounds, many of which are included in our dataset. For example, the X_2_VAl Heusler series featured in this manuscript, with X-sites choices Sc, Cu, Y, Nb, Mo, Hf, Ta and W, are not included in the MP website.

**Fig. 1:**
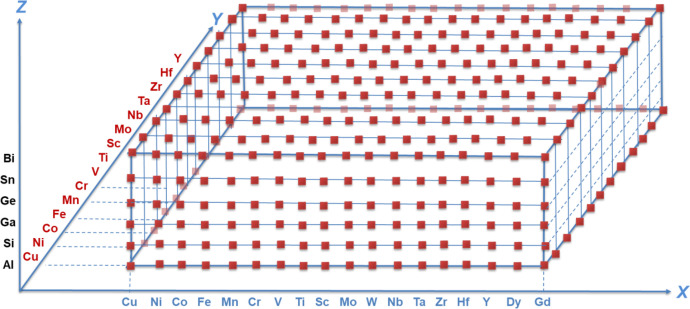
Orthogonal element choice for density functional theory calculations.

For our calculation, we have selected 18 D-block (including 2 rare earth) elements for X-site, most of these elements have also been used as Y-site and 6 main group elements as Z-site. Fig. 1 shows the orthogonal element choices for this work, sorted outward by atomic radius. Our calculations are based on a four atom-based face-centered cubic unit cell with 2 X-sites atoms and 1 Y and 1 Z site atoms, which represents one formula unit. Full Heusler structures consist of four interpenetrating face-centered cubic (fcc) sublattices positioned at (0, 0, 0), (1/4, 1/4, 1/4), (1/2, 1/2, 1/2), and (3/4, 3/4, 3/4). The sublattice sites (0, 0, 0) and (1/2, 1/2,1/2) are occupied by X atoms in a regular Heusler. The remaining sublattice sites (1/4, 1/4, 1/4) and (3/4,3/4, 3/4) are occupied by Y and Z elements respectively. 423 out of more than a thousand combinations have been calculated and presented in this dataset.

**Fig. 2:**
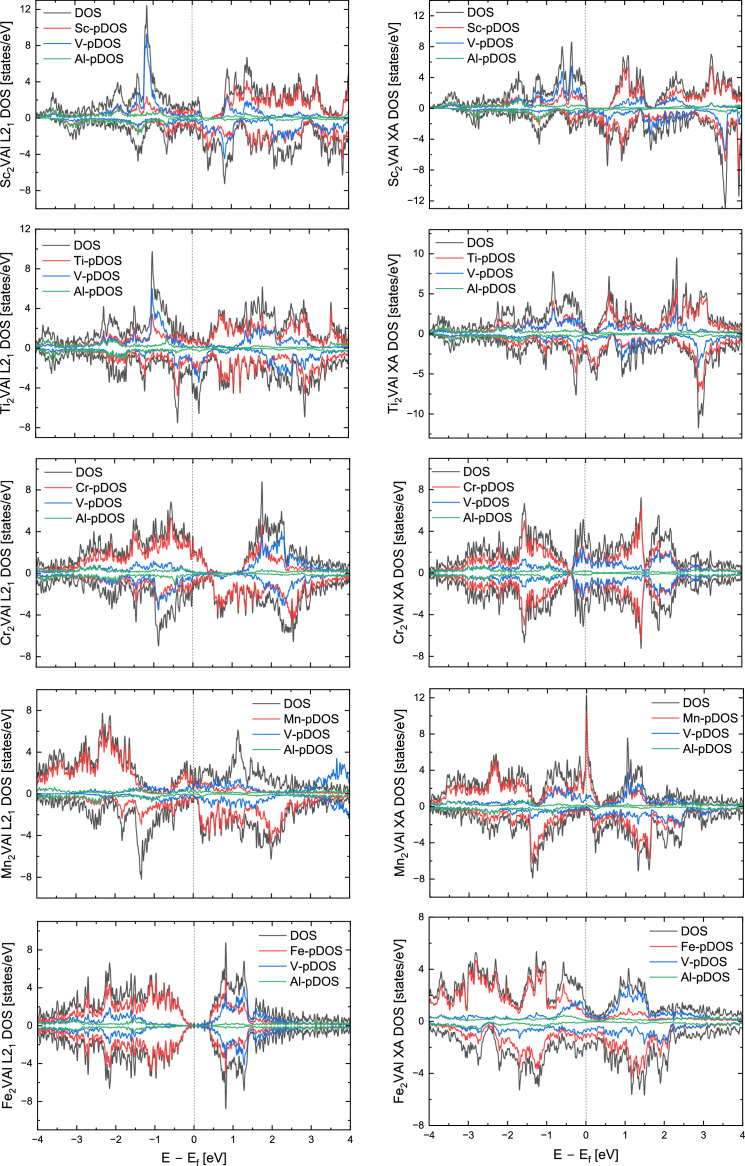

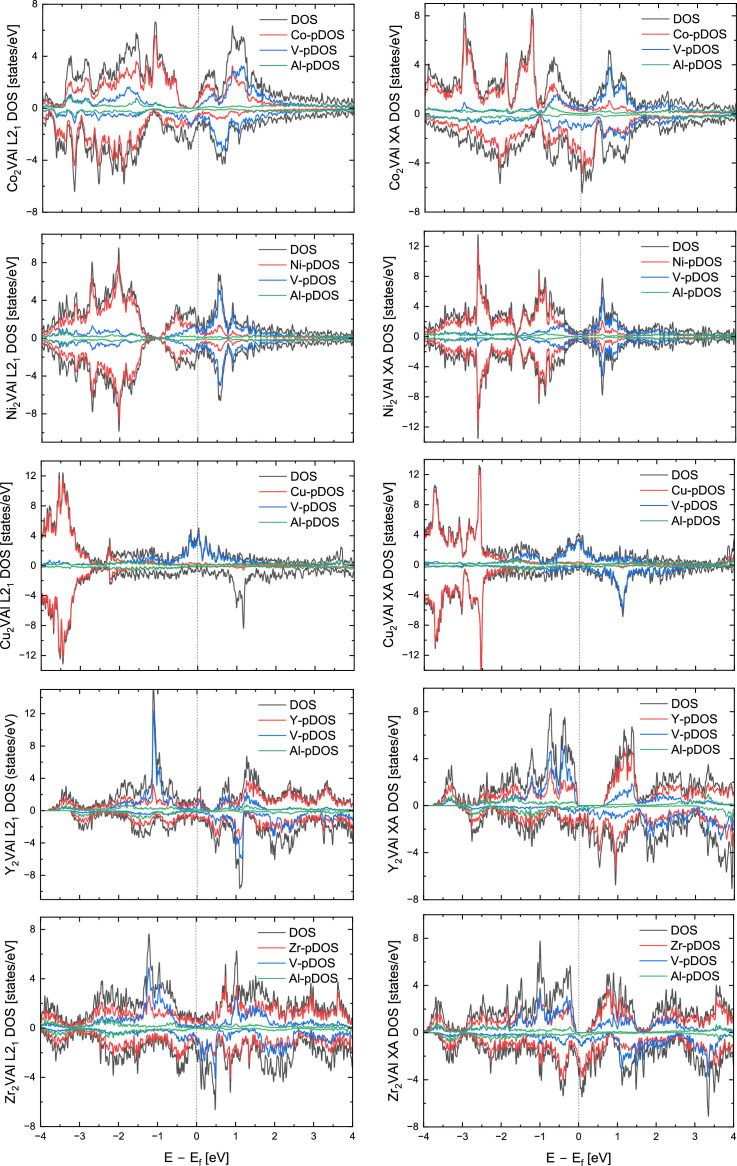

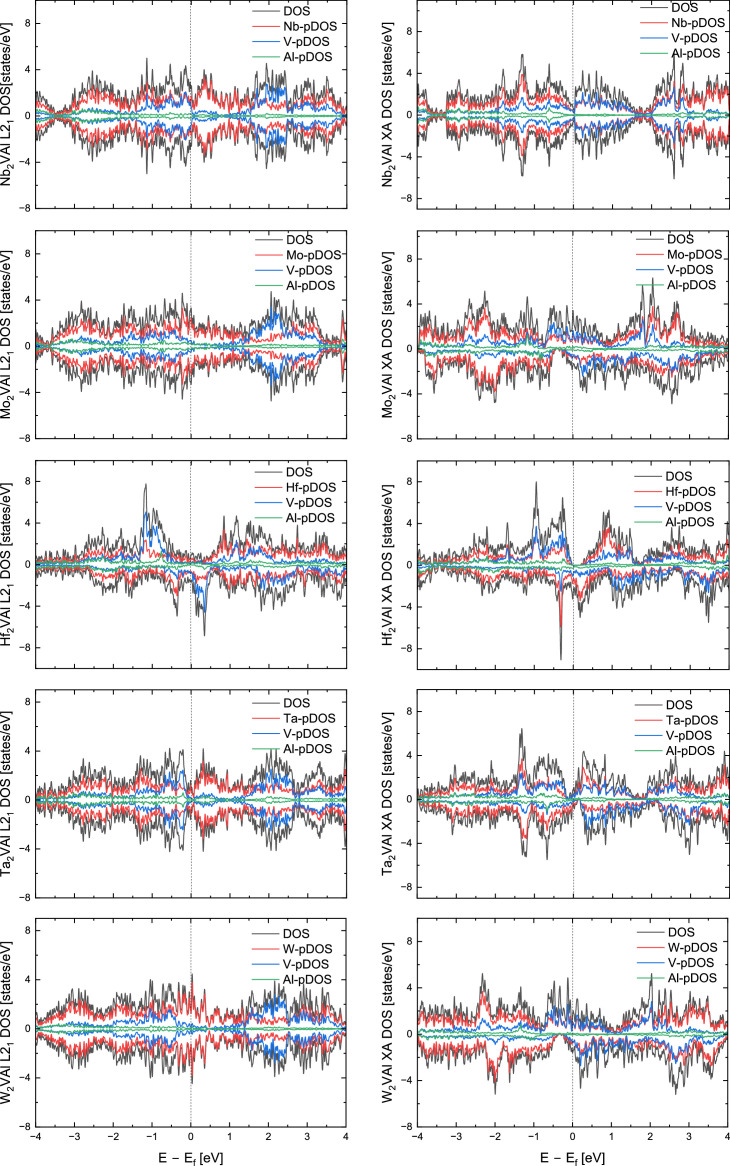
Total density of states (DOS) and projected density of states (pDOS) of *X_2_*VAl Heusler alloys (*X* = Sc, Ti, Cr, Mn, Fe, Co, Ni, Cu, Y, Zr, Nb, Mo, Hf, Ta, W).

Table 1 summarizes the data collected on a series of *X*_2_VAl (*X* = Sc, Ti, Cr, Mn, Fe, Co, Ni, Cu, Y, Zr, Nb. Mo, Hf, TA, W) full (L2_1_) and inverse (XA) Heusler alloys. The total energy of the system can be conceptualized as the energy required to construct the system at absolute zero temperature by assembling single atoms of constituting elements. The calculations of density of states show that Y_2_VAl, Zr_2_VAl and Hf_2_VAl are half metals in their inverse Heusler phases. Fig. 2 represents the total and atom-resolved density of states of *X*_2_VAl alloy series in their L2_1_ and XA phases.

**Table 1:** A list of lattice constant, energy, magnetic moment, and spin polarization of *X*_2_VAl Heusler alloy in their regular (*L2_1_*) and inverse (*XA*) Heusler phases.

Alloy	Phase	Lattice constant Å	Total energy (Ry)	Formation energy (eV/atom)	Magnetic moment per site, *μ_B_*				Total magnetic moment, *μ_B_/f.u.*	Spin polarization
					*X_1_*	*X_2_*	*Y*	*Z*		
Sc_2_VAl	L2_1_	6.7714	−567.4977	−0.17056	0.1673	0.1673	2.3806	−0.0714	3.23	5.23%
	XA	6.7489	−567.4663	−0.06391	0.0825	0.4922	2.4785	−0.0323	3.86	43.53%
Ti_2_VAl	L2_1_	6.3407	−620.9777	−0.19732	−0.0287	−0.0286	1.255	−0.0288	1.4	50.73%
	XA	6.3417	−620.9583	−0.13104	−0.3327	0.546	1.3756	−0.0156	1.96	70.68%
Cr_2_VAl	L2_1_	5.9524	−749.9959	−0.08087	1.6235	1.6236	−0.7581	−0.0033	2.71	43.51%
	XA	5.8951	−749.9738	−0.00545	0	0	0.0001	0	0	0.00%
Mn_2_VAl	L2_1_	5.9690	−826.6618	−0.37786	1.559	1.5583	−1	−0.026	2	25.23%
	XA	5.9015	−826.5904	−0.13511	2.5428	0.5262	0.7166	−0.0218	3.98	94.91%
Fe_2_VAl	L2_1_	5.7099	−912.2007	−0.69231	0	0	0.0001	0	0	0%
	XA	5.7741	−911.9523	0.15255	1.6024	1.8661	−0.2875	−0.0047	3.46	38.87%
Co_2_VAl	L2_1_	5.7594	−1007.034	−0.33519	−0.9443	−1.0985	−0.1302	0.0284	−2	25.23%
	XA	5.7988	−1006.981	−0.15652	0.8335	1.4253	−0.3906	−0.0079	1.76	67.46%
Ni_2_VAl	L2_1_	5.8068	−1111.966	−0.38555	−0.0069	0.0117	0.0041	−0.0004	0.01	6.09%
	XA	5.7966	−1111.937	−0.28638	0	0.0001	−0.0002	0	0	0%
Cu_2_VAl	L2_1_	5.9579	−1227.323	−0.07506	0.0224	0.0224	1.2526	−0.033	1.41	85.28%
	XA	5.9684	−1227.302	−0.00293	−0.0089	0.0455	1.3728	0.0362	1.63	69.85%
Y_2_VAl	L2_1_	7.2090	−818.7558	−0.08784	0.1014	0.1014	2.6523	−0.0912	3.3	37.36%
	XA	7.1527	−818.699	0.10459	0.0417	0.3554	2.78	−0.0385	4	100%
Zr_2_VAl	L2_1_	6.7401	−867.999	−0.16669	−0.0432	−0.0432	1.6678	−0.0454	1.75	40.55%
	XA	6.7089	−867.9606	−0.03418	−0.2972	0.1226	1.8524	−0.006	2	100%
Nb_2_VAl	L2_1_	6.4164	−922.0781	−0.05676	0	0	0	0	0	0%
	XA	6.4071	−922.0882	−0.09100	0	0	0	0	0	0%
Mo_2_VAl	L2_1_	6.2018	−981.3697	−0.03651	0.0003	0.0003	−0.0004	0	0	0%
	XA	6.2290	−981.3879	−0.09844	0.15	−0.1405	1.2328	0.0102	1.49	37.71%
Hf_2_VAl	L2_1_	6.6621	−1646.007	−0.14042	−0.022	−0.022	1.5501	−0.0477	1.68	24.50%
	XA	6.6488	−1645.964	0.00859	−0.1961	0.1817	1.6867	−0.0242	2	100%
Ta_2_VAl	L2_1_	6.4010	−1707.811	−0.03086	0.0055	−0.0055	0	0	0	0%
	XA	6.4143	−1707.816	−0.04591	−0.4263	−0.1553	0.8536	−0.0172	0.08	31.81%
W_2_VAl	L2_1_	6.2154	−1774.013	0.12564	0.0001	0.0001	−0.0001	0	0	0%
	XA	6.2514	−1774.051	−0.00202	0.1607	−0.0885	1.2538	0.016	1.63	0.00%

The total density of states (DOS) and site-specific projected density of states (pDOS) of *X*_2_VAl where *X* is one of the fifteen 3d elements presented in Table 1 are shown in Fig. 2. The L2_1_ phases are displayed on the left and the *XA* phases are on the right side of the figure. The *X*-pDOS is the sum of two *X* atoms in the alloy. The Fermi energy (*E_F_*) is shifted to zero and is marked by a vertical line.

**List of all included alloys, organized by Y-Z combination.**

**X_2_FeAl:** Sc_2_FeAl, Ti_2_FeAl, V_2_FeAl, Cr_2_FeAl, Mn_2_FeAl, Co_2_FeAl, Ni_2_FeAl, Cu_2_FeAl, Y_2_FeAl, Zr_2_FeAl, Nb_2_FeAl, Mo_2_FeAl, Hf_2_FeAl, Ta_2_FeAl, W_2_FeAl, Dy_2_FeAl, Gd_2_FeAl
**X_2_FeGe:** Sc_2_FeGe, Ti_2_FeGe, V_2_FeGe, Cr_2_FeGe, Mn_2_FeGe, Co_2_FeGe, Ni_2_FeGe, Cu_2_FeGe, Y_2_FeGe, Zr_2_FeGe, Nb_2_FeGe, Hf_2_FeGe, Ta_2_FeGe, W_2_FeGe, Dy_2_FeGe, Gd_2_FeGe
**X_2_FeSi:** Sc_2_FeSi, Ti_2_FeSi, V_2_FeSi, Cr_2_FeSi, Mn_2_FeSi, Co_2_FeSi, Ni_2_FeSi, Cu_2_FeSi, Y_2_FeSi, Zr_2_FeSi, Hf_2_FeSi, Ta_2_FeSi, W_2_FeSi, Gd_2_FeSi, Dy_2_FeSi
**X_2_FeGa:** Sc_2_FeGa, Ti_2_FeGa, V_2_FeGa, Cr_2_FeGa, Mn_2_FeGa, Ni_2_FeGa, Cu_2_FeGa, Y_2_FeGa, Zr_2_FeGa, Ta_2_FeGa, Gd_2_FeGa
**X_2_FeBi:** Cr_2_FeBi
**X_2_FeSn:** Cr_2_FeSn
**X_2_TiAl:** Sc_2_TiAl, V_2_TiAl, Cr_2_TiAl, Mn_2_TiAl, Fe_2_TiAl, Co_2_TiAl, Ni_2_TiAl, Cu_2_TiAl, Y_2_TiAl, Zr_2_TiAl, Nb_2_TiAl, Mo_2_TiAl, Hf_2_TiAl, Ta_2_TiAl, W_2_TiAl, Gd_2_TiAl, Dy_2_TiAl
**X_2_TiSi:** Sc_2_TiSi, V_2_TiSi, Cr_2_TiSi, Mn_2_TiSi, Fe_2_TiSi, Co_2_TiSi, Ni_2_TiSi, Cu_2_TiSi, Y_2_TiSi, Zr_2_TiSi, Nb_2_TiSi, Mo_2_TiSi, Hf_2_TiSi, Ta_2_TiSi, W_2_TiSi, Gd_2_TiSi, Dy_2_TiSi
**X_2_TiGe:** Sc_2_TiGe, V_2_TiGe, Cr_2_TiGe, Mn_2_TiGe, Fe_2_TiGe, Co_2_TiGe, Ni_2_TiGe, Cu_2_TiGe, Y_2_TiGe, Zr_2_TiGe, Nb_2_TiGe, Mo_2_TiGe, Hf_2_TiGe, Ta_2_TiGe, W_2_TiGe, Gd_2_TiGe, Dy_2_TiGe
**X_2_TiGa:** V_2_TiGa, Cr_2_TiGa, Mn_2_TiGa, Fe_2_TiGa, Co_2_TiGa, Ni_2_TiGa, Cu_2_TiGa, Zr_2_TiGa, Ta_2_TiGa, W_2_TiGa, Gd_2_TiGa, Dy_2_TiGa
**X_2_TiSn:** Co_2_TiSn
**X_2_YAl:** Sc_2_YAl, Ti_2_YAl, V_2_YAl, Cr_2_YAl, Mn_2_YAl, Fe_2_YAl, Co_2_YAl, Ni_2_YAl, Cu_2_YAl, Zr_2_YAl, Nb_2_YAl, Hf_2_YAl, Ta_2_YAl, W_2_YAl, Gd_2_YAl, Dy_2_YAl
**X_2_YGe:** Sc_2_YGe, Ti_2_YGe, V_2_YGe, Cr_2_YGe, Mn_2_YGe, Fe_2_YGe, Co_2_YGe, Ni_2_YGe, Cu_2_YGe, Zr_2_YGe, Nb_2_YGe, Hf_2_YGe, Ta_2_YGe, W_2_YGe, Gd_2_YGe, Dy_2_YGe
**X_2_YSi:** Sc_2_YSi, Ti_2_YSi, V_2_YSi, Cr_2_YSi, Mn_2_YSi, Fe_2_YSi, Co_2_YSi, Ni_2_YSi, Cu_2_YSi Zr_2_YSi, Nb_2_YSi, Hf_2_YSi, Ta_2_YSi, W_2_YSi, Gd_2_YSi, Dy_2_YSi
**X_2_YGa:** Sc_2_YGa, Ti_2_YGa, Cr_2_YGa, Mn_2_YGa, Fe_2_YGa, Co_2_YGa, Ni_2_YGa, Cu_2_YGa, Zr_2_YGa, Nb_2_YGa, Hf_2_YGa, Ta_2_YGa, W_2_YGa, Gd_2_YGa
**X_2_YBi:** Sc_2_YBi, Ti_2_YBi, V_2_YBi, Cr_2_YBi, Mn_2_YBi, Fe_2_YBi, Co_2_YBi, Ni_2_YBi, Cu_2_YBi, Zr_2_YBi, Nb_2_YBi, Mo_2_YBi, Hf_2_YBi, Ta_2_YBi, W_2_YBi, Dy_2_YBi, Gd_2_YBi
**X_2_YSn:** Sc_2_YSn, Ti_2_YSn, V_2_YSn, Cr_2_YSn, Mn_2_YSn, Fe_2_YSn, Co_2_YSn, Ni_2_YSn, Cu_2_YSn, Zr_2_YSn, Nb_2_YSn, Mo_2_YSn, Hf_2_YSn, W_2_YSn, Ta_2_YSn
**X_2_CuAl:** Sc_2_CuAl, Cr_2_CuAl, Mn_2_CuAl, Fe_2_CuAl, Co_2_CuAl, Ni_2_CuAl, Nb_2_CuAl, Hf_2_CuAl
**X_2_CuSi:** Cr_2_CuSi, Mn_2_CuSi, Fe_2_CuSi, Co_2_CuSi, Ni_2_CuSi
**X_2_CuGa:** Sc_2_CuGa, Ti_2_CuGa, V_2_CuGa, Cr_2_CuGa, Mn_2_CuGa, Fe_2_CuGa, Co_2_CuGa, Ni_2_CuGa, Y_2_CuGa, Zr_2_CuGa, Nb_2_CuGa, Mo_2_CuGa, Hf_2_CuGa, Ta_2_CuGa, W_2_CuGa, Dy_2_CuGa, Gd_2_CuGa
**X_2_CuGe:** Sc_2_CuGe, Ti_2_CuGe, V_2_CuGe, Cr_2_CuGe, Mn_2_CuGe, Fe_2_CuGe, Co₂_2_CuGe, Ni_2_CuGe, Y_2_CuGe, Zr_2_CuGe, Nb_2_CuGe, Mo_2_CuGe, Hf_2_CuGe, Ta_2_CuGe, W_2_CuGe, Dy_2_CuGe, Gd_2_CuGe
**X_2_CuSn:** Mn_2_CuSn
**X_2_CuSn:** Mn_2_CuBi
**X_2_CoAl:** Sc_2_CoAl, Ti_2_CoAl, V_2_CoAl, Cr_2_CoAl, Mn_2_CoAl, Fe_2_CoAl, Ni_2_CoAl, Cu_2_CoAl, Y_2_CoAl, Zr_2_CoAl, Nb_2_CoAl, Mo_2_CoAl, Hf_2_CoAl, Ta_2_CoAl, W_2_CoAl, Dy_2_CoAl, Gd_2_CoAl
**X_2_CoGa:** Ti_2_CoGa, Cr_2_CoGa Fe_2_CoGa, Ni_2_CoGa, Gd_2_CoGa, Dy_2_CoGa
**X_2_CoGe:** Cr_2_CoGe, Mn_2_CoGe, Fe_2_CoGe, Ni_2_CoGe, Gd_2_CoGe, Dy_2_CoGe
**X_2_CoSi:** Cr_2_CoSi, Mn_2_CoSi, Fe_2_CoSi, Ni_2_CoSi, Cu_2_CoSi, Mo_2_CoSi, Gd_2_CoSi, Dy_2_CoSi
**X_2_CoBi:** Cr_2_CoBi, V_2_CoBi
**X_2_CoSn:** Cr_2_CoSn, V_2_CoSn
**X_2_MnAl:** Sc_2_MnAl, Cr_2_MnAl, Fe_2_MnAl, Co_2_MnAl, Ni_2_MnAl, Cu_2_MnAl, Nb_2_MnAl, Hf_2_MnAl
**X_2_MnSi:** Sc_2_MnSi, Ti_2_MnSi, V_2_MnSi, Cr_2_MnSi, Fe_2_MnSi, Co_2_MnSi, Ni_2_MnSi, Cu_2_MnSi
**X_2_MnGe:** Sc_2_MnGe, Cr_2_MnGe, Co_2_MnGe, Ni_2_MnGe
**X_2_MnGa:** Cr_2_MnGa, Co_2_MnGa, Ni_2_MnGa
**X_2_MnSn:** Co_2_MnSn
**X_2_MnBi:** Co_2_MnBi
**X_2_CrAl:** Sc_2_CrAl, Fe_2_CrAl, Mn_2_CrAl, Ni_2_CrAl, Nb_2_CrAl, Hf_2_CrAl, Dy_2_CrAl, Gd_2_CrAl
**X_2_CrSi:** Fe_2_CrSi, Ni_2_CrSi, Gd_2_CrSi, Dy_2_CrSi
**X_2_CrGa:** Ni_2_CrGa, Gd_2_CrGa, Dy_2_CrGa
**X_2_CrGe:** Ni_2_CrGe, Gd_2_CrGe, Dy_2_CrGe
**X_2_NiAl:** Sc_2_NiAl, Cr_2_NiAl, Cu_2_NiAl, Nb_2_NiAl, Hf_2_NiAl, Ta_2_NiAl
**X_2_NiSi:** Cr_2_NiSi
**X_2_NiGa:** Cr_2_NiGa
**X_2_NiGe:** Cr_2_NiGe
**X_2_NiBi:** Cr_2_NiBi
**X_2_NiSn:** Cr_2_NiSn
**X_2_ScAl:** Cr_2_ScAl, Fe_2_ScAl, Ni_2_ScAl, Cu_2_ScAl, Nb_2_ScAl, Hf_2_ScAl
**X_2_ScSi:** Cr_2_ScSi, Fe_2_ScSi, Ni_2_ScSi
**X_2_ScGa:** Cr_2_ScGa, Fe_2_ScGa, Ni_2_ScGa
**X_2_ScGe:** Cr_2_ScGe, Fe_2_ScGe, Ni_2_ScGe
**X_2_ScSn:** Co_2_ScSn
**X_2_ZrAl:** Cr_2_ZrAl, Fe_2_ZrAl, Ni_2_ZrAl, Nb_2_ZrAl
**X_2_ZrSi:** Cr_2_ZrSi, Fe_2_ZrSi, Ni_2_ZrSi
**X_2_ZrGa:** Cr_2_ZrGa, Fe_2_ZrGa, Ni_2_ZrGa
**X_2_ZrGe:** Cr_2_ZrGe, Fe_2_ZrGe, Ni_2_ZrGe
**X_2_VAl:** Sc_2_VAl, Ti_2_VAl, Cr_2_VAl, Mn_2_VAl, Fe_2_VAl, Co_2_VAl, Ni_2_VAl, Cu_2_VAl, Y_2_VAl, Zr_2_VAl, Nb_2_VAl, Mo_2_VAl, Hf_2_VAl, Ta_2_VAl, W_2_VAl
**X_2_VGa:** Cr_2_VGa, Fe_2_VGa, Ni_2_VGa
**X_2_VGe:** Cr_2_VGe, Fe_2_VGe, Ni_2_VGe
**X_2_VSi:** Cr_2_VSi, Fe_2_VSi, Co_2_VSi, Ni_2_VSi
**X_2_NbAl:** Fe_2_NbAl, Y_2_NbAl, Hf_2_NbAl, Ta_2_NbAl
**X_2_TaAl:** Hf_2_TaAl
**X_2_HfGe:** Co_2_HfGe
**X_2_HfAl:** Co_2_HfAl
**X_2_MoAl:** Fe_2_MoAl

## 4 Experimental Design, Materials and Methods

For the calculation, we have created four atom-based face-centered cubic unit cell and calculated its structural, electrical, and magnetic properties in both regular and inverse Heusler phases. It's worth mentioning here that inverse Heusler phase is a disordered structure of the full Heusler phase and can be obtained by swapping one of the *X*-site atoms with the *Y*-site. The density functional equations were solved using the plane-wave pseudopotential and projector-augmented-wave (PAW) approaches. The unknown exchange-correlation energy functional within the Khon-Sham equation was parametrized within the Perdew-Bruke-Ernzerhof (PBE) version of the generalized gradient approximation GGA. A Monkhorst-Pack special k-point mesh of 20 × 20 × 20, covering the irreducible part of the Brillouin zone was used for k-space integration. In our calculation, we didn't optimize cutoff energies, instead, we used very high cutoff to minimize error. The wave-function cutoff was set to 250 Ry for all alloy and element calculations and the kinetic energy cutoff for charge density for all cases were four times the wave-function cutoff i.e., 1000 Ry. (1 Ry = 13.6057 eV). The convergence of the total energy to a minimum value of 10–9 Ry was set as a criterion for the convergence of self-consistency loop and force convergence cutoff 10^−5^ Ry was used for all alloys and element calculations. The equilibrium lattice constant of these compounds was obtained by fitting the quadratic energy-volume graph, utilizing the Birch-Murnaghan equation of state.

(1) $$E_{T}\left(V\right)=\;E_{T}\;\left(V_{0}\right)+\;\frac{B_{0}V}{B_{0}^{\prime }}\;\left[\frac{{\left(V_{0}/V\right)}^{B_{0}^{\prime }}}{B_{0}^{\prime }-1}+1\right]-\;\frac{V_{0}B_{0}}{B_{0}^{\prime }-1}$$

Where *E_T_* is the total energy for given volume *V, B_0_* is the bulk modulus at the equilibrium volume *V_0_* and B′_0_ is the pressure derivative of the bulk modulus at the equilibrium volume *V_0_*. The ground state lattice structure was obtained by relaxing the system using conjugate-gradient method which allows the cell shape and volume change freely until it finds the ground-state. All alloys were considered spin-polarized and a small non-zero starting magnetization value was set for all atomic types in the alloy. Pseudopotentials from Quantum Espresso PSLibrary (generated by A. Dal Corso) [13] were used in our calculations and are included in the Supplementary Material. The formation energy of the alloys is calculated by subtracting the energy corresponding to individual element from the calculated total energy (*E_total_*) of the alloy. The value of *E_total_* was obtained from the ground state self-consistent (SCF) calculations.

(2) $$\Delta E_{form\;}=\;E_{total}^{X_{2}YZ}-2*\;E_{total}^{X}-E_{total}^{Y}-\;E_{total}^{Z}$$

For elemental calculations, the calculation steps and convergence parameters were the same as what we used for alloy calculations. The known ground state structure was used for all elements, e.g., simple hexagonal structure for Scandium, Hafnium, Zirconium, bcc structure for Chromium, Iron, fcc structure for Copper, Silicon, Aluminum, etc. A non-SCF calculation was performed after the self-consistent field (SCF) calculation to get the density of states (DOS) and projected density of states (pDOS) of each of these alloys. Spin Polarization ($P_{F})$ was calculated by using the density of states (DOS) at the Fermi energy in spin-up and -down states.

(3) $$P_{F}=\;\left|\frac{n_{E_{F}}^{↑}-n_{E_{F}}^{↓}\;}{n_{E_{F}}^{↑}+n_{E_{F}}^{↓}}\right|\times \;100\%$$

The calculations were performed without considering the effect of spin-orbit coupling.

## Limitations

None.

## CRediT authorship contribution statement

**Ridwan Nahar:** Conceptualization, Investigation, Data curation, Writing – original draft. **Ka Ming Law:** Investigation, Data curation. **Thomas Roden:** Investigation, Data curation. **Michael Zengel:** Investigation, Data curation. **Justin Lewis:** Investigation, Data curation. **Sujan Budhathoki:** Investigation, Data curation. **Riley Nold:** Investigation, Data curation. **Harshil Avlani:** Investigation, Data curation. **Babajide Akintunde:** Investigation, Data curation. **Naomi Derksen:** Investigation, Data curation. **Adam J. Hauser:** Conceptualization, Validation, Data curation, Writing – review & editing, Project administration, Funding acquisition.

## Data Availability

Dataset on Density Functional Theory investigation of Ternary Heusler Alloys (Original data) (Mendeley Data)
